# Effect of π-spacer moieties coupled to a porphyrin/PC70BM donor–acceptor for promising organic photovoltaic properties: a DFT study

**DOI:** 10.1039/d6ra01296e

**Published:** 2026-03-25

**Authors:** Malik Muhammad Asif Iqbal, Fakhar Hussain, Hina Javeed, Talha Hassan, Riaz Hussain

**Affiliations:** a Department of Chemistry, University of Okara Okara-56300 Pakistan fakharsakhi@gmail.com mmasif101@gmail.com

## Abstract

The present study involved the design of porphyrin-based molecules (FF1–FF4) with a D1-π-D2-π-D1 configuration. This was accomplished by adding distinct π-spacers to a reference donor molecule. This research employs DFT and time-dependent DFT analyses at the CAM-B3LYP/6-31G(d,p) level to explore optoelectronic and photovoltaic characteristics. The designed molecules showed a decrease in the band gap, with values ranging from 3.86 to 4.07 eV, compared to R (4.13 eV). Furthermore, the molecules displayed bathochromic shifts in the visible region in the gas phase (419.13–435.58 nm) and in the chlorobenzene phase (442.41–457.89 nm) relative to R (410.82 and 436.41 nm, respectively). Moreover, evidence of intramolecular charge transfer (ICT) was obtained by employing DOS and TDM graphical maps. The presence of naphthalene groups at the terminus of the porphyrin core may facilitate charge mobility. FF1 has been proven to be the most promising candidate, exhibiting exceptional photovoltaic properties. Among these characteristics are a minimum band gap of 3.86 eV, extremely low reorganization energies for electrons (*λ*_e_ = 0.0030 eV) and holes (*λ*_h_ = 0.0022 eV), maximum absorption wavelengths of 435.58 nm in gas phase and 457.89 nm in chlorobenzene, and a preferred open-circuit voltage (*V*_OC_) of 1.59 eV in comparison to the PC70BM LUMO acceptor. All of these characteristics are present. The results show that the molecules under investigation are good candidates for organic solar cell (OSC) applications as donor materials and hole-transport materials, and they perform very well in photovoltaic studies.

## Abstract 2

The present study involved the design of porphyrin-based molecules (FF1–FF4) with a D1-π-D2-π-D1 configuration.

## Graphical abstract

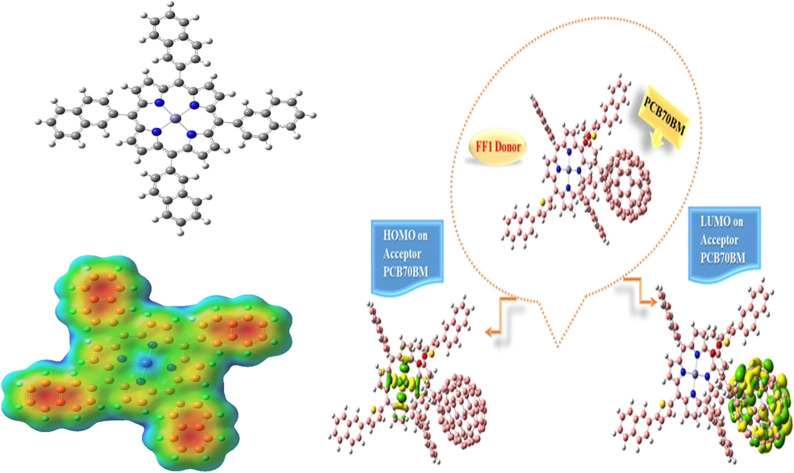

## 1 Introduction

In the contemporary global context, the most significant challenge pertains to the identification of sustainable energy sources, the cost implications of their deployment, and the associated drawbacks of their utilization. The prevailing concern is to address the energy crisis through the utilization of cost-effective energy sources, given the prevailing reliance on traditional, non-renewable energy sources that contribute to environmental concerns, including the greenhouse effect and air pollution.^1^ To surmount these issues, researchers are exploring new and more reliable sources and techniques for energy production, including wind mills, energy from biomass, and organic and perovskite solar cells.^2^ Solar cells are a particularly compatible source of energy conversion, with the capacity to convert sunlight into electricity. This electricity can then be utilized for both domestic and industrial purposes, thereby addressing the global demand for energy.^3^ Solar-powered workplaces, industries, and rooftops have been shown to attract people's attention and increase reliance on solar energy. The solar cells operate on the principle of the photovoltaic effect, which converts sunlight into electric energy.^4,5^

Now the world is moving towards the use and synthesis of organic and perovskite solar cells, as these are more reliable, extraordinarily efficient, available in a wide range of varieties, lightweight, and have customizable energy levels.^6,7^ The design of donor–acceptor (D/A) systems represents a particularly intriguing area of research, as these systems hold significant potential for applications in artificial photosynthesis and organic solar cells.^8–13^ Polymer solar cells (PSCs) employing porphyrin donors and fullerene acceptors have attracted significant research attention due to their potential for sustainable and renewable energy production.^14–20^ However, since porphyrin molecules disperse uncontrollably throughout the polymer thin films, prior efforts to integrate porphyrin into PSCs have had little success. We need new approaches to have porphyrin-fullerene complexes dispersed in polymer thin films. Synthesis and characterization of porphyrin end-functionalized polymers with well-defined, regulated polymer chain topologies are the primary focus of this study.^21,22^ Using fullerene derivatives with porphyrin-functionalized polymers demonstrates that porphyrin/fullerene donor–acceptor layers can be tuned to self-assemble into polymer thin films. The strong π–π interactions between the fullerene and porphyrin moieties, along with the good compatibility between the polymer backbone and the fullerene component, are the causes of this behaviour. It is very difficult and usually requires very complex synthetic methods to covalently incorporate porphyrin molecules into polymer chains.^20,23^ The subsequent area of interest was the regulated self-assembly of a porphyrin/fullerene donor–acceptor complex (layer) utilizing a combination of polymers, fullerenes, and free porphyrin molecules. New studies show that using metal-based substances such as porphyrins or phthalocyanine to create interfacial dipole layers that realign energy levels and prevent defect states from forming is useful.^24,25^

This work presents the synthesis of four novel porphyrin-based compounds (FF1–FF4) for potential use in future photovoltaic devices by incorporating π-spacer moieties into the reference molecule (*R*). The end-capped structures exhibit a D1-π-D2-π-D1 arrangement in the newly developed frameworks. Furthermore, the used π-spacers served as conduits for charge transfer from the electron-donating region to the electron-accepting region. We examined the donor–acceptor interaction between the Porphyrin donor and the PC70BM acceptor to elucidate their electrical and optoelectronic properties in organic solar cells. The molecular structure of both parts is very important for determining how well charge transfer and excitation occur. Porphyrin is a good electron donor because it has a long π-conjugated framework and readily absorbs light. PC70BM is a good electron acceptor because it has a high electron affinity and can readily accept electrons.

In this study, density functional theory (DFT) and time-dependent density functional theory (TD-DFT) were utilized to investigate critical factors, including frontier molecular orbitals (FMOs), density of states (DOS), light-harvesting efficiency, photovoltaic performance, ionization potential, electron affinity, transition density matrix (TDM), quantum-chemical parameters, electron–hole analysis, reorganization energies (*λ*_e_ and *λ*_h_), excitation energies, optical properties of the engineered materials, and optical absorption qualities. The results obtained provide valuable insights into the mechanisms of electron movement and charge transfer between donor and acceptor molecules. This finding indicates that the porphyrin/PC70BM system is well-suited for solar energy applications. A comprehensive study has been conducted to evaluate the mobility of charges from the donor component to the acceptors. The study demonstrates that the proposed molecules may be used to fabricate effective photovoltaic systems.

We used density functional theory (DFT) and time-dependent density functional theory (TD-DFT) to investigate critical factors such as frontier molecular orbitals (FMOs), density of states (DOS), light-harvesting efficiency, photovoltaic performance, ionization potential, electron affinity, transition density matrix (TDM), quantum-chemical parameters, electron–hole analysis, reorganization energies (*λ*_e_ and *λ*_h_), excitation energies, optical properties of the engineered materials and optical absorption qualities. These results shed light on how electrons move and how charge flows between the donor and acceptor molecules. This shows that the porphyrin/PC70BM system is suitable for efficient solar applications. A comprehensive study evaluates the mobility of charges from the donor component to the acceptors, demonstrating that the proposed molecules may be used to fabricate effective photovoltaic systems.

## 2 Computational methodology

All computations were conducted with Gaussian 09.,^26^ and the Gauss View 5.0 application was used to display the results.^27^ Time-dependent (TD-DFT) computations were conducted for the reference chemical R using five functionals (B3LYP,^28^ CAM-B3LYP,^29^ MPW1PW91,^30^ M06 (ref. 31) and M06-2X^32^ with a 6-31G (d,p) basis set to model the absorption spectra. The reference molecule's (*R*) predicted *λ*_max_ was compared with the relevant experimental data in order to verify the theoretical approach. We used the CAM-B3LYP functional with the 6-31G(d,p) basis set for all subsequent computations because it best agreed with the experimental result among those we evaluated.

The maximum values of the investigated compounds were determined in both the gas phase and the chlorobenzene-solvated state. The Integral Equation Formalism Polarizable Continuum Model (IEFPCM)^33^ employed to analyze the influence of the solvent. The Origin 6.0 software was utilized for spectral mapping. The CAM-B3LYP/6-31G(d,p) theoretical level was used to determine charge transfer, reorganization energies, frontier orbitals, and DOS. The same function was used to calculate the Mulliken charges for all the suggested compounds. The CAM-B3LYP/6-31G(d,p) theoretical level was also used to figure out the reorganization energies of all the suggested compounds (FF1–FF4), along with the reference *R*. There were two main types of reorganization energy: internal reorganization energy (*λ*_int_.) and outer reorganization energy (*λ*_ext_). The energy of internal reorganization is about making quick changes to the internal structure, while the energy of external reorganization is about calming the outside world. We focused only at the internal reorganization energy in this study and neglected to look at how external environmental factors affected it. So, the eqn (1) & (2) (ref. 34) are used to figure out the reorganization energies of the electron (*λ*_e_) and the hole (*λ*_h_).

1 *λ*_h_ = (*E*_0_^+^ − *E*_+_) + (*E*_0_^+^ − *E*_0_)

2 *λ*_e_ = (*E*_0_^−^ − *E*^−^) + (*E*_0_^−^ − *E*_0_)

The energy of the neutral molecule, as defined by the optimized geometry of the anion and cation, is represented as *E*_−_^0^ and *E*_+_^0^*E*_+_ and *E*_−_ show the energies of the cation and anion after the neutral molecules have been optimized. *E*_0_^+^, and *E*_0_^−^ show the single point energy of the cation and anion of the neutral molecule has been optimized in a basic way. *E*_0_ is the singular point energy of the neutral molecule in its fundamental state.

## 3 Results and discussion

**Fig. 1:**
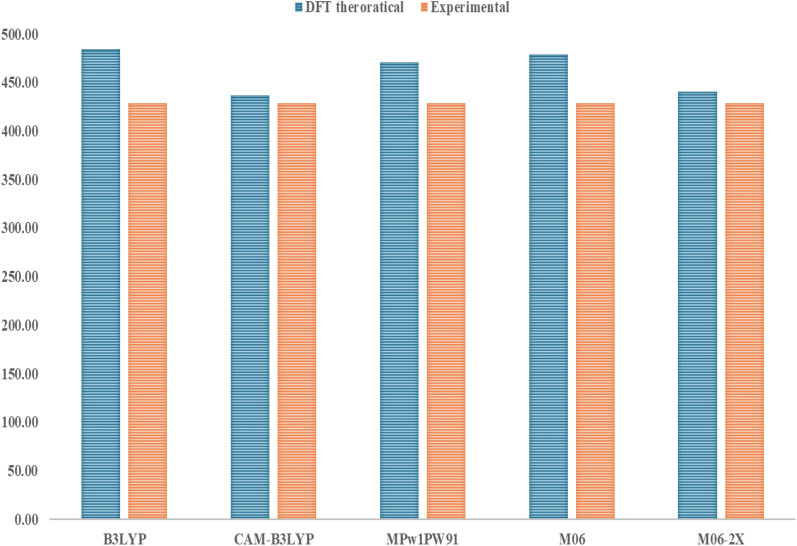
Comparison of R values calculated using six different density functionals in a chlorobenzene solvent.

In this work, different functionals were used, such as B3LYP, CAM-B3LYP, MPW1PW91, M06, and M06-2x, along with a 6-31G(d,p) basis set to look at the optoelectronic properties of the chemical R. Fig. 1 shows the comparison between the experimental value of 429 nm and the theoretical *λ*_max_ values of the reference molecule *R*, which were calculated using different DFT functionals. Based on its results, CAM-B3LYP/6-31G(d,p) is the best approach to employ for future computations, since it closely matches the experimental data with a *λ*_max_ of 436.41 nm.

**Fig. 2:**
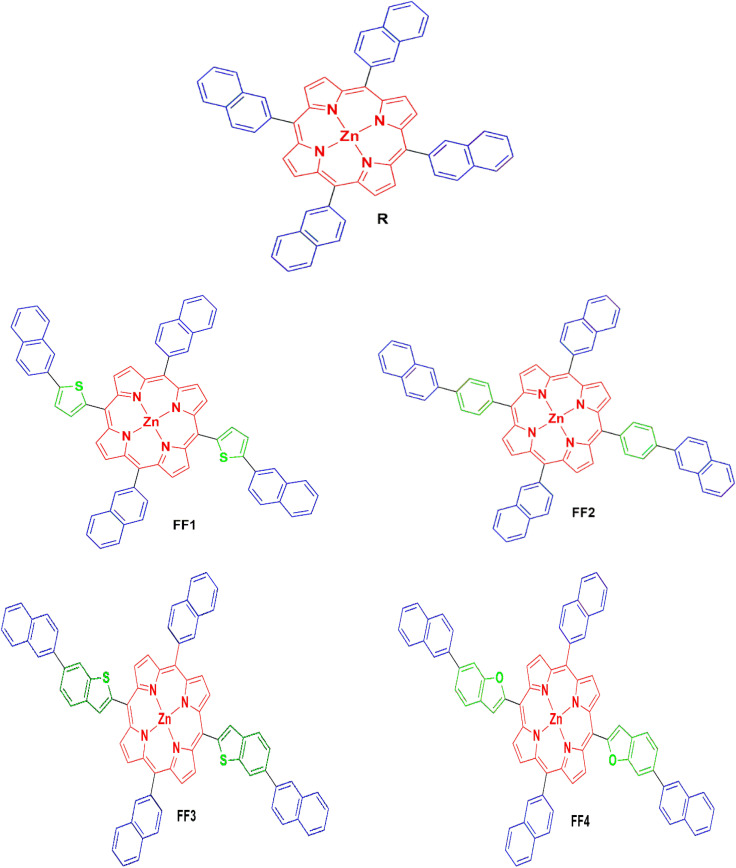
Optimized molecular structures of the reference (R) and designed molecules (FF1–FF4).

**Fig. 3:**
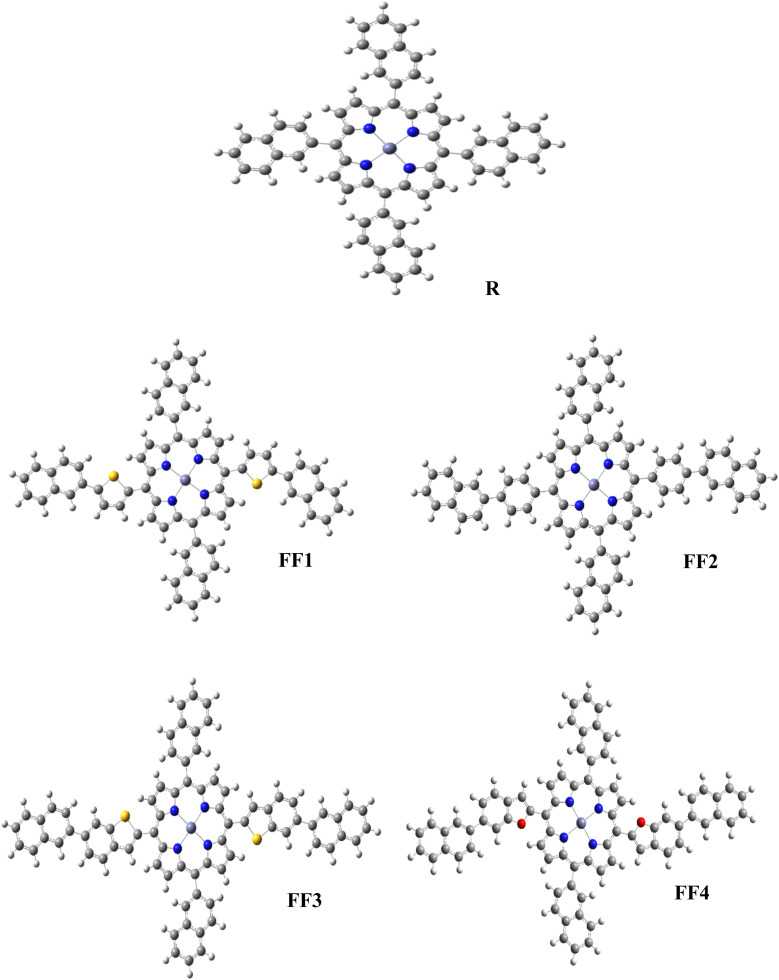
Systematically optimized structure of the reference R and the created molecules FF1–FF4.

Fig. 2 shows the molecular arrangements of the reference R and the established compounds (FF1–FF4). Fig. 3 illustrates the optimized geometry of R and the developed molecules (FF1–FF4) at the CAM-B3LYP/6-31G(d,p) basis set level.

### 3.1 FMOs analysis

**Fig. 4:**
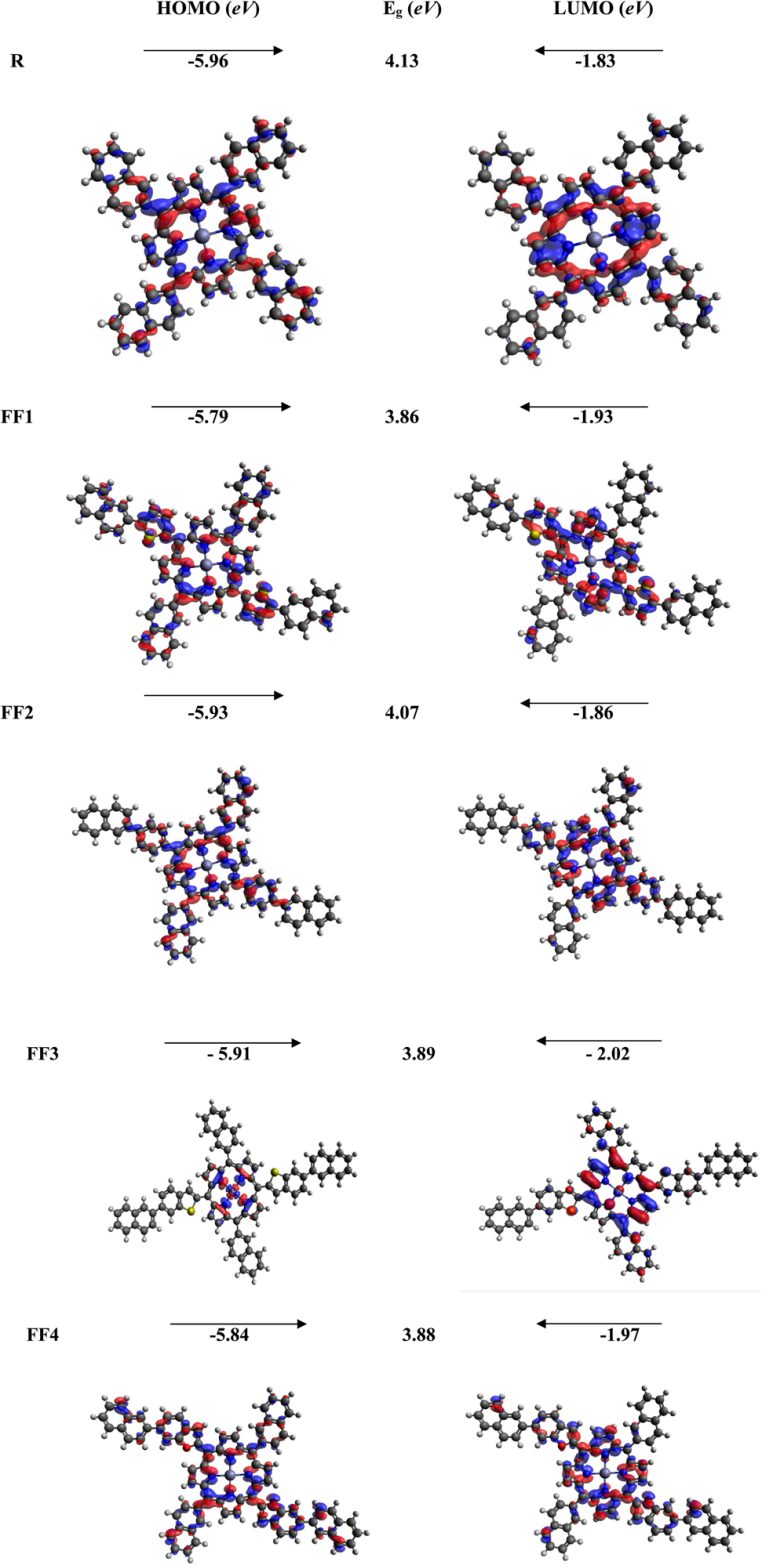
Calculated HOMO–LUMO spatial distribution patterns of R and FF1–FF4.

In the optimized geometries, the donor unit, π-bridge, and acceptor moiety are coplanar, indicating a highly planar, structurally homogeneous molecular framework.^35,36^ Frontier molecular orbital (FMO) study is essential for comprehending the optoelectronic properties of organic semiconductors (OSCs). Fig. 4 displays the FMO diagrams of all examined compounds, depicting the spatial distribution of electron density in the highest occupied molecular orbital (HOMO) and the lowest unoccupied molecular orbital (LUMO). The primary objective of the molecular design approach is to enhance conjugation and promote effective charge delocalization in the donor–acceptor system through the incorporation of π-spacers. The incorporation of π-spacers is imperative, as they serve to modify the electronic communication between the core and the terminal acceptor units, thereby exerting a substantial influence on the molecules' electronic structure. The electron density is distributed along the conjugated backbone, particularly around the electron-withdrawing acceptor units, as these pi-spacers facilitate extended π-conjugation, rather than being confined to a single central site. Consequently, the site preference energy was not examined directly; rather, the electronic properties were analyzed by means of an examination of the distributions of frontier molecular orbitals and charge-transfer characteristics. These factors delineate the energetically favourable locations for electron localization. The outcomes demonstrate that the π-spacer method effectively stabilizes the electron density towards the acceptor regions and promotes efficient intramolecular charge transfer, thereby validating the relevance of the chosen molecular design strategy. At the CAM-B3LYP/6-31G(d,p) theoretical level, the reference molecule displays HOMO and LUMO energy levels of −5.96 eV and −1.83 eV, respectively. The synthesized compounds FF1, FF2, FF3, and FF4 exhibit HOMO values of −5.79, −5.93, −5.91, and −5.84 eV, with corresponding LUMO energies of −1.93, −1.86, −2.02, and −1.97 eV, respectively. The difference in energies between the HOMO and LUMO (eqn (3)) is called the energy gap (*E*_g_). It is a key factor in how charges move between molecules.^37–39^

3 *E*_LUMO_ − *E*_HOMO_ = *E*_g_

The charge-transfer rate increases in proportion to the decrease in the energy gap. To the best of our knowledge, each of the molecules that we have developed has a smaller energy gap in comparison to the reference R. Because of this, the molecules that we created exhibit superior opto-electronic characteristics compared to R. All of the molecules, FF1, FF2, FF3, and FF4, had energy gaps between their HOMO and LUMO states that are around 3.86 eV, 4.07 eV, 3.89 eV, and 3.88 eV, respectively. Among all of the developed molecules (FF1, FF2, FF3, and FF4), including reference R (4.13 eV), FF1 has the smallest discrepancy between its HOMO and LUMO values.

### 3.2 Optical properties

**Fig. 5:**
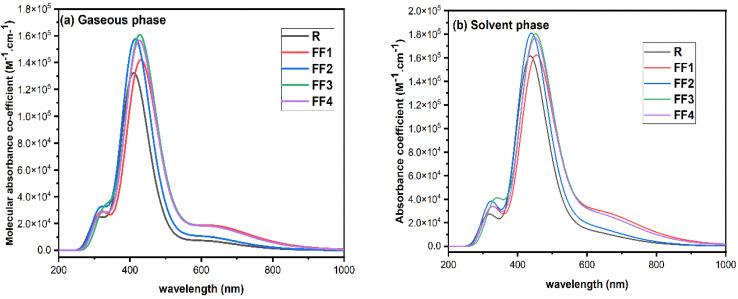
UV-vis spectra of R and all suggested molecules (FF1–FF4) in the gaseous phase and chloroform solvent.

The photovoltaic (PV) efficiency of molecules may be greatly increased by adjusting their electrical and optical properties. A broad absorption spectrum, a high light-harvesting capacity, and low excitation energy are necessary for organic solar cells' (OSCs') best performance.^40^ Both near-infrared and visible spectrums show individual peaks in the structure's significant absorption.^41,42^ All calculations were performed at the TD-DFT CAM-B3LYP/6-31G(d,p) theoretical level in the gas phase to ascertain the absorption spectra of the proposed molecules (FF1–FF4), which include the reference component R. The maximum absorption (*λ*_max_), *E*_x_, oscillator strength (*f*_os_), and work in the gaseous phase are presented in Table 1. From 419.13 nm to 435.58 nm, the values of *λ*_max_ are found to be within the range. The *λ*_max_ value of reference R is obtained to be 410.82 nm, while the *λ*_max_ values of all the designed molecules, namely FF1, FF2, FF3, and FF4, are determined to be 435.58 nm, 419.13 nm, 434.18 nm, and 433.54 nm, respectively. The compound FF1 shows the highest absorption at 435.58 nm and the lowest energy gap of 3.86 eV among all other molecules. This is attributed to the presence of highly π-spacer elements. In terms of rising *λ*_max_ values, the order of created compounds is as follows: FF1 > FF3 > FF4 > FF2 > R. This sequence corresponds to the decreasing order of band gap energies among the compounds. A representation of the simulated absorption spectra of FF1–FF4 and reference (R) at the TD-DFT CAM-B3LYP/6-31G(d,p) level can be seen in Fig. 5.

**Table 1:** Experimental and theoretical calculations of *λ*_max_, *E*_x_, and *f*_os_ for R and all proposed compounds (FF1–FF4) in the gas phase

Molecules	DFT *λ*_max_ (nm)	Exp *λ*_max_ (nm)	*E*_x_ (eV)	*f*_os_	Major MO assignment
R	410.82	429	3.018	1.6296	HOMO → LUMO (43%)
FF1	435.58	—	2.846	2.1197	HOMO → LUMO (65%)
FF2	419.13	—	2.958	2.0712	HUMO → LUMO (60%)
FF3	434.18	—	2.856	2.1700	HUMO → LUMO (65%)
FF4	433.54	—	2.860	2.2687	HOMO → LUMO (64%)

The UV-visible absorption spectra of the designed molecules (FF1–FF4) and the standard compound R are also calculated at the CAM-B3LYP/6-31G(d,p) level in chlorobenzene, the liquid phase. There is information about the assignment, the oscillator strength (*f*), the excitation energy (eV), and the highest absorption (*λ*_max_) in Table 2. This information is gathered in the solvent step. The presence of π-spacer moieties clearly changes the value of *λ*_max_ when the region of 442.41 nm to 457.89 nm is analyzed; the absorption spectra of FF1–FF4 can be found. When evaluated in the gas phase, the *λ*_max_ of all molecules in the solvent may be higher. R, the reference chemical, has a maximum wavelength of 436.41 nanometers. Every one of the created molecules has a red shift in their absorption spectra when related to R. Each of the developed compounds has an absorption maximum that follows the following order: FF1 > FF3 > FF4 > FF2 > R. In Fig. 5, the absorption spectra of every molecule that is in the solvent phase are shown. Taking into consideration the information presented above, it is possible to employ all four proposed molecules (FF1–FF4) as donor molecules. However, FF1 is the most suitable candidate for synthesis and testing in photovoltaic systems. The *E*_x_ (excitation energy) is the energy needed to transfer an electron between different energy level orbitals.^43^

**Table 2:** Experimental and theoretically determined *λ*_max_, *E*_x_, *f*_os_, reference R, and all designed compounds (FF1–FF4) in chlorobenzene solvent

Molecules	DFT *λ*_max_ (nm)	Exp. *λ*_max_ (nm)	*E*_x_ (eV)	*f*_os_	Major MO assignment
R	436.41	429	2.841	1.9915	HUMO → LUMO (32%)
FF1	457.89	—	2.708	2.2609	HOMO → LUMO (73%)
FF2	442.41	—	2.802	2.2832	HOMO → LUMO (65%)
FF3	455.76	—	2.720	2.263	HOMO → LUMO (70%)
FF4	455.74	—	2.720	2.3879	HOMO → LUMO (69%)

### 3.3 DOS analysis

**Fig. 6:**
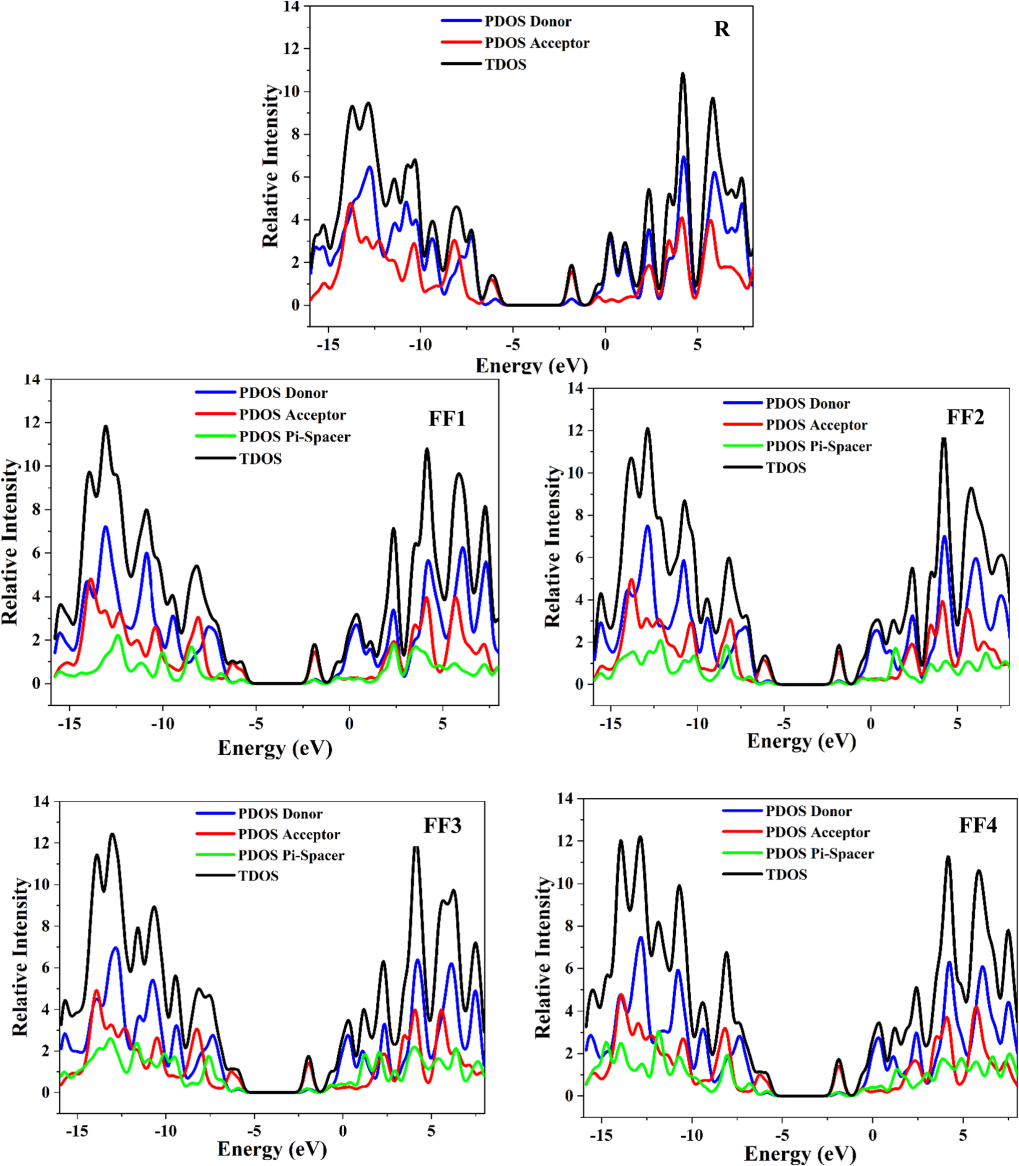
PDOS and TDOS of donor, acceptor, and pi-spacer for the reference R and the designed compounds FF1–FF4.

A partial density of states (PDOS) study of the synthesized compounds (FF1–FF4) and reference R was carried out at the CAM-B3LYP/6-31G(d,p) to augment the FMO diagram (Fig. 4). The density-of-states (DOS) spectra for all substances are shown in Fig. 6. The pi-bridging group clearly alters the distribution of the HOMO and LUMO. The HOMO electron density of compound R is mostly found at the donor–acceptor border and the bridge region. Furthermore, the LUMO density is mostly located within the molecule and the pi-bridging groups. In FF1, the HOMO density is uniformly distributed throughout the entire molecule, whereas the lowest unoccupied molecular orbitals (LUMOs) are symmetrically divided between the acceptor group and the pi-bridging group.^44^

The structure of FF4's orbitals is very similar to that of FF1's. The highest occupied molecular orbitals (HOMOs) are widely distributed across the entire molecular framework. The LUMOs on the other hand, are mostly localized on the acceptor and π-bridge units, with the donor moiety contributing less. For all investigated molecules, the HOMOs are largely localized on the acceptor unit, whereas the LUMOs are mainly spread over both the donor and acceptor segments, along with the π-bridging region. FF3 exhibits a distinct orbital distribution behavior, in which both the HOMO and LUMO are strongly localized within the molecular core. In comparison, FF2 shows a broader delocalization of the LUMO across the π-bridging unit, while FF3 demonstrates only limited LUMO distribution in this region.

The percentages of each component in the HOMO and LUMO states are shown in Table 3, demonstrating that electron transport occurs from the core to the terminal acceptors. A percentage value of % indicates that the terminal acceptors of FF3 contribute the most to the LUMO. Because of the precise position of their molecular structure, acceptor molecules exhibit conductive properties that depend on their location. When the molecule is in closer proximity to the LUMO state, the availability of a greater number of electronic states makes it possible for acceptors to play a more substantial role in the electric conduction process. As a consequence of the pi-spacer groups that were created, the LUMO peaks in FF1–FF4 have been elevated, which provides evidence that terminal group modification is an effective method for increasing the conductivity of acceptor molecules when they are paired with a donor that is compatible with them.

**Table 3:** DFT-based analysis of percentage donor and acceptor in elevating the HOMO and LUMO of R and FF1–FF4

Molecule	Excitation energy state	% Age contribution donor (eV)	% Age contribution pi-spacer (eV)	% Age contribution acceptor (eV)
R	HOMO	—	—	—
	LUMO	—	—	—
FF1	HOMO	19.2	20.1	60.7
	LUMO	5.9	17.0	77.2
FF2	HOMO	19.4	13.1	67.5
	LUMO	6.9	10.9	82.2
FF3	HOMO	17.1	22.0	60.9
	LUMO	3.5	18.1	78.4
FF4	HOMO	17.0	24.4	58.7
	LUMO	4.1	15.9	80.0

### 3.4 TDM analysis

Through the use of transition density matrix (TDM) research, one can understand the behavior of the acceptor and donor components throughout the framework. More particularly, one can gain insight into the capacity of excitons to escape from the coulombic forces within the framework. Electronic transitions between donor and acceptor fragments in a molecule can be determined using TDM analysis, which provides a method for this. When establishing the types of charge localization and delocalization within the molecule under consideration, these plots play a vital role. Therefore, TDM analysis makes it easier to characterize the exciton density at different places across the molecule. Because hydrogen atoms have a negligible influence on transition characteristics, these TDM graphs were automatically omitted from consideration.

**Fig. 7:**
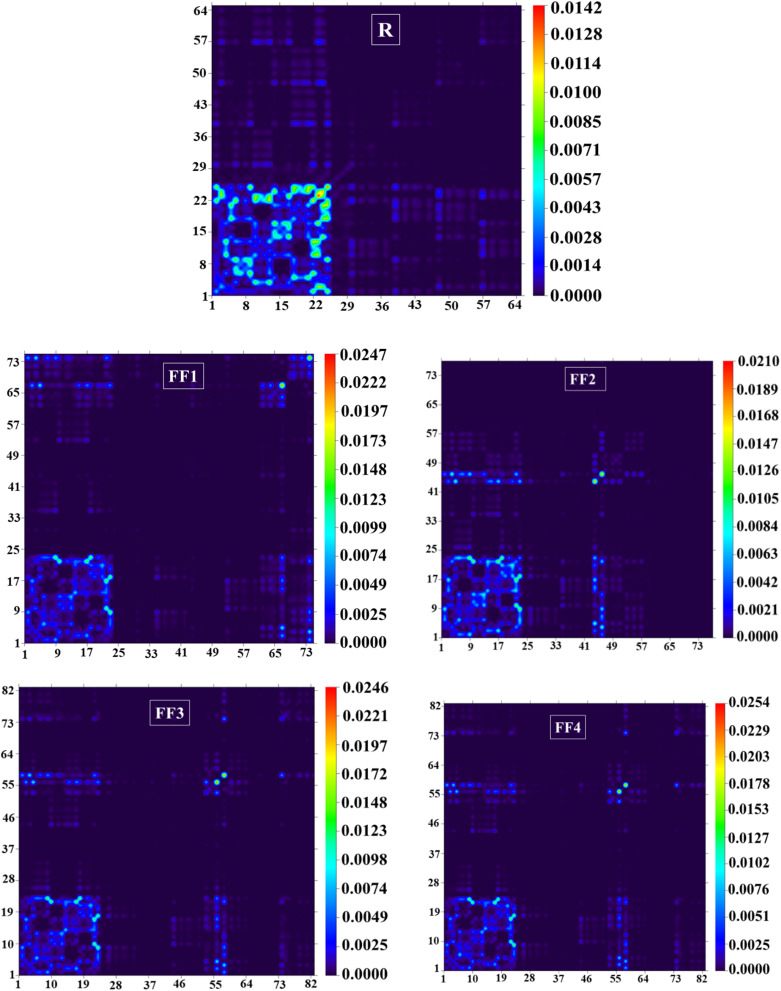
TDM graphs of the reference (R) and the developed compounds (FF1–FF4).

To make understanding electronic excitations easier, the number of atoms in each molecule was split into two independent components in these plots. These components are referred to as the donor (D) and the acceptor (A). A uniform exciton distribution from donor to acceptor groups is found in the majority of the molecules that were studied, as shown in Fig. 7. This observation provides evidence that the charge is effectively transferred from donor to acceptor units. Additionally, the presence of numerous bright spots in the acceptor and donor regions of the investigated molecules strongly implies a high charge density in these regions. In conclusion, it is possible to assert that the pi-bridging moieties provide a major contribution to the accomplishment of the effective transfer of excitons from the donor to the acceptor. An in-depth analysis of the TDM graphs indicates that excitons are moving appreciably across the structures under investigation. This migration will transport excitons to the donor/acceptor interface, where they dissociate into electrons and holes, facilitating efficient charge transfer throughout the process. The observed exciton migrations are especially noticeable in the structures developed, designated FF1–FF4.

### 3.5 Global reactivity parameters

To predict the chemical reactivity of substances, global reactivity parameters are used. These parameters are derived by quantum techniques. The energies of the HOMO and LUMO molecular orbitals govern the ionization potential (IP), electron affinity (EA), chemical potential (*µ*), hardness (*η*), and softness (*S*). These values are shown in Table 4. All of the compounds developed, numbered FF1 through FF4, have a chemical potential more negative than R, indicating they are electron acceptors. In order to estimate all of the parameters, the following were calculated by using eqn (4)–(11).^45–48^

4 IP = (*E*_0_^+^ − *E*_0_)

5 EA = (*E*_0_ − *E*_0_^−^)

6
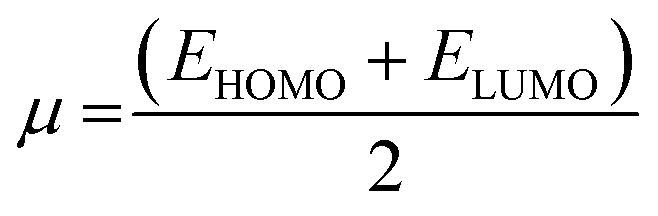
7
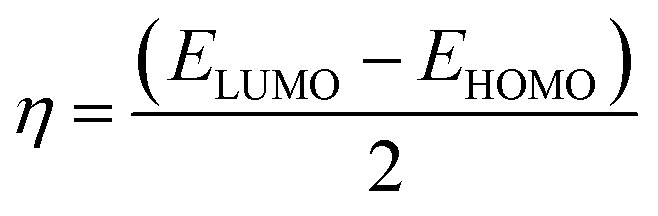
8
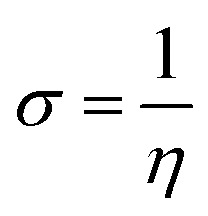
9
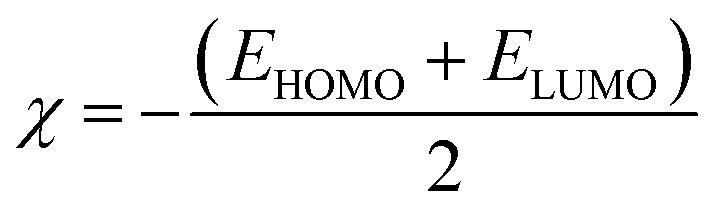
10
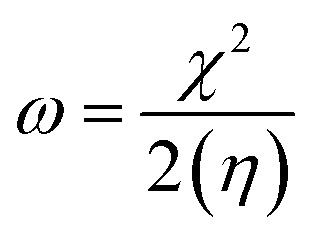
11
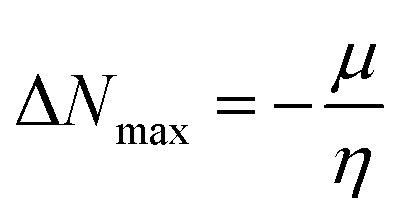


**Table 4:** Computed electronic parameters of the reference molecule R and designed compounds FF1–FF4, providing insights into their frontier molecular orbital energies and chemical reactivity

Molecules	IP (eV)	EA (eV)	*µ* (eV)	*η* (eV)	*S* (eV)	*X* (eV)	*ω* (eV)	Δ*N*_max_ (eV)
R	6.01	1.69	−4.81	1.15	0.87	3.89	8.70	4.18
FF1	5.82	1.85	−4.83	0.96	1.04	3.86	7.17	5.02
FF2	5.99	1.77	−4.97	0.93	1.07	3.96	7.31	5.34
FF3	5.93	1.96	−4.97	0.93	1.07	3.96	7.31	5.34
FF4	5.88	1.88	−4.89	0.95	1.05	3.91	7.28	5.12

Materials having a low ionization potential facilitate the removal of electrons and the generation of charge carriers, resulting in enhanced charge mobility. Thus, these materials may enhance the performance of solar devices. The ionization potentials of R and the design compounds (FF1–FF4) are 0.214, 0.220, 0.218, 0.216, and 0.221 eV, respectively. The adiabatic ionization potential increases in the following order: FF1 < FF4 < FF3 < FF2 < R. The results suggest that FF3 has the lowest IP among all the compounds. This signifies that it can donate electrons more quickly than R, hence possessing enhanced charge transfer capabilities. Materials with elevated electron affinity exhibit enhanced charge mobility, since the addition of an electron to form a charge carrier is facilitated. Device performance could be improved as a result.

**Fig. 8:**
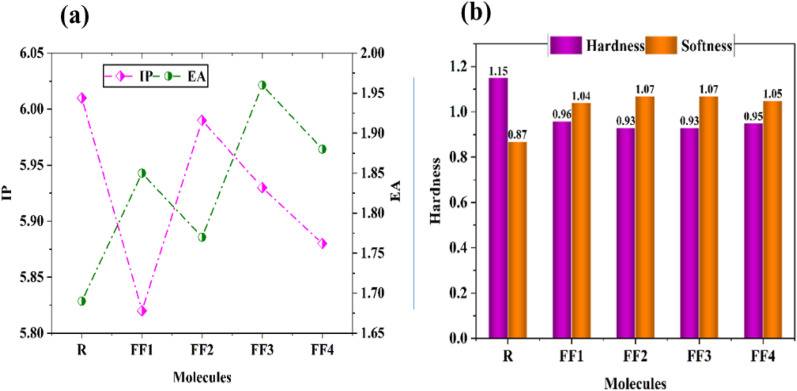
(a) Ionization potential (IP) and electron affinity (EA), (b) hardness and softness of R and designed molecules (FF1–FF4).

The calculated chemical hardness values for the designed molecules (0.93–0.96 eV) are slightly lower than that of R (1.15 eV). However, it is evident that the stability of the molecule is within an acceptable range, as all the values are positive. The reduced hardness is indicative of enhanced charge-transfer capability and elevated molecular polarizability. The properties of organic photovoltaic materials are enhanced by these effects, which allow for enhanced electronic communication within the conjugated framework and efficient donor–acceptor charge separation. The proposed molecules display favourable electrical properties for solar applications and maintain adequate stability, as evidenced by the obtained hardness values. In intramolecular charge transfer, charges can be more readily transferred from the donor to the acceptor area when the acceptor has a high electron affinity. Organic solar cells (OSCs) are more efficient because charges are more mobile, which improves charge transfer. R and the newly formed compounds (FF1–FF4) had electron affinity values of 0.068, 0.065, 0.072, 0.069, and 0.062 eV, respectively. The elevated electron affinities seen in Fig. 8 demonstrate that all the modified compounds possess an enhanced charge-attracting capability.

The chemical potential represents the energetic orientation of an electron within a system. This may be associated with electronegativity, in which a more negative chemical potential indicates greater difficulty in electron loss but greater ease in electron acquisition. The designed compounds (FF1–FF4) exhibit lower chemical potentials than R, validating their electron-accepting properties.

### 3.6 Heat map

**Fig. 9:**
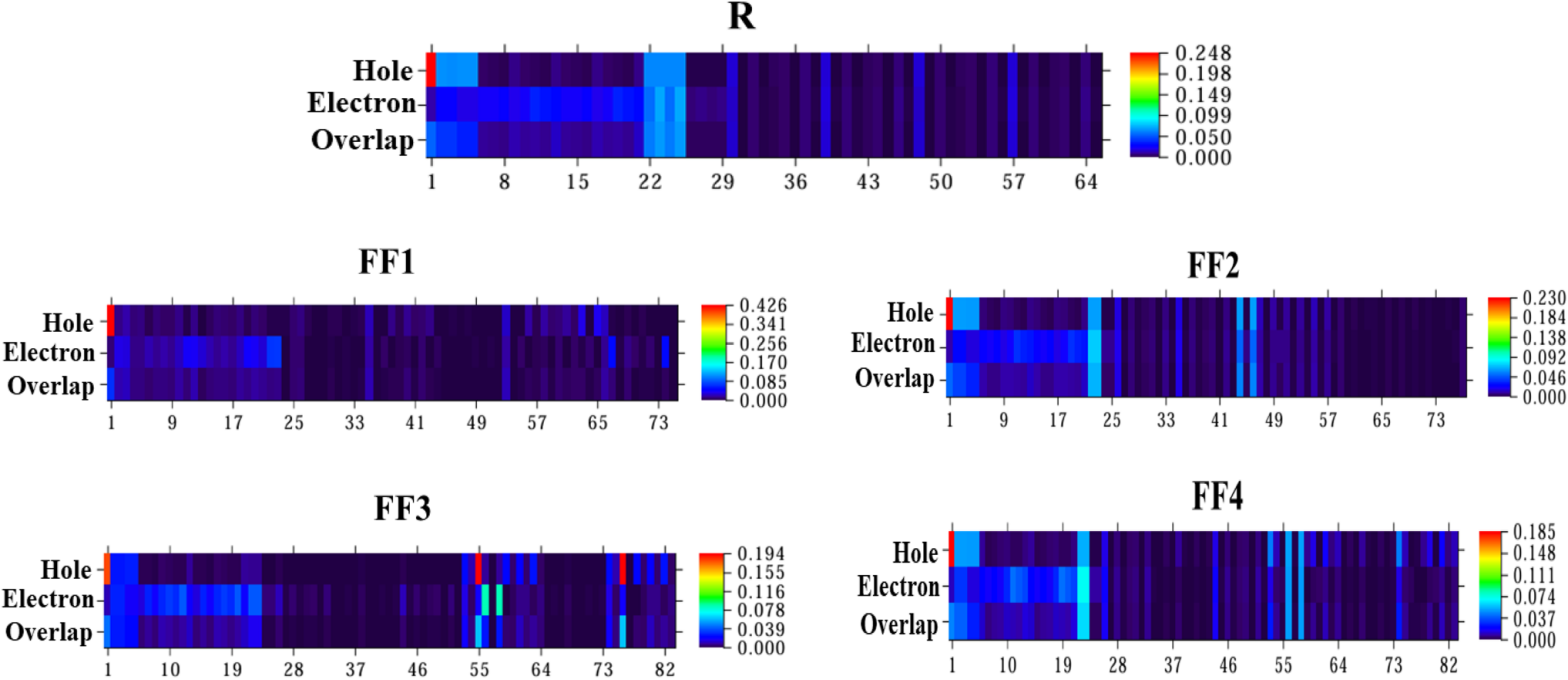
Heat map representations of hole and electron contributions for R and FF1–FF4.

Applying hole–electron contribution maps is a good way to guess how charges will move between different parts of the same molecule. The atoms involved in forming holes and electrons are shown on the heat maps. In addition, it shows how holes and electrons interact. It is not possible to include hydrogen atom effects in heat maps because these atoms are not involved in any significant electronic changes. Each engineered molecule is split into a donor and an acceptor so that the contributions from different parts can be more clearly seen. The iso-surface maps were generated with Multiwfn using the CAM-B3LYP/6-31G(d, p) functional, as shown in Fig. 9.

The heat maps for each molecule feature numerous non-hydrogen atoms along the *x*-axis. In contrast, a colour scale is displayed on the *y*-axis, indicating values that represent contributions to hole–electron overlap and hole–electron contribution. The red regions depicted in these iso-surface maps signify the extent of hole contribution. The principal source of the hole arises from the carbon atoms of the acceptor units that connect the donor and acceptor moieties.

### 3.7 Reorganization energy

Reorganization energy (RE) profoundly influences the efficiency of organic solar cells (OSCs). It enables the evaluation of charge-carrier mobility within the compound. There is an inverse relationship between the molecular reorganization energy (RE) and the charge-transfer mobilities of electrons and holes. Because of this, lower RE values are linked to better electron (*λ*_e_) and hole (*λ*_l_) movement. Thus, the theoretical RE values for all the compounds examined are shown in Table 5. The findings of this research suggest that RE qualities are comparable to the compound's molecular planarity.

**Table 5:** Hole (*λ*_h_) and electron (*λ*_e_) reorganization energies (eV) for the reference R and designed compounds FF1–FF4

Compounds	*λ*_e_ (eV)	*λ*_h_ (eV)
R	0.0054	0.0022
FF1	0.0030	0.0031
FF2	0.0036	0.0021
FF3	0.0033	0.0027
FF4	0.0037	0.0026

It can be seen that the electron reorganization energy (*λ*_e_) decreases in the following order: FF4 > FF2 > FF3 > FF1. The fact that all the designed compounds have lower reorganization energies than the standard molecule R suggests they could be good materials for electron transport. Better exciton dissociation and improved electron mobility are enabled by these substances' favorable molecular geometry and planar arrangement. It can be seen that the electron reorganization energy (*λ*_e_) decreases in the following order: FF4 > FF2 > FF3 > FF1. The fact that all the designed compounds have lower reorganization energies than the standard molecule R suggests they could be good materials for electron transport. Better exciton dissociation and improved electron mobility are enabled by these substances' favorable molecular geometry and planar arrangement.

The low RE value (eV) for electron transmission in FF3 indicates that this molecule is suitable as an effective electron transport material in all OSCS.

A comparable pattern arises for the RE values of holes (*λ*_h_), showing the same tendency observed for electron values. Consequently, FF1 is not a markedly better hole-transporting material owing to its somewhat elevated hole values when juxtaposed with the R molecule. Although all other molecules exhibit commendable *λ*_h_ transport characteristics, FF2 and FF4 are the most promising candidates in their respective photovoltaic cells and may facilitate the development of efficient organic solar cells.

### 3.8 MEP analysis

**Fig. 10:**
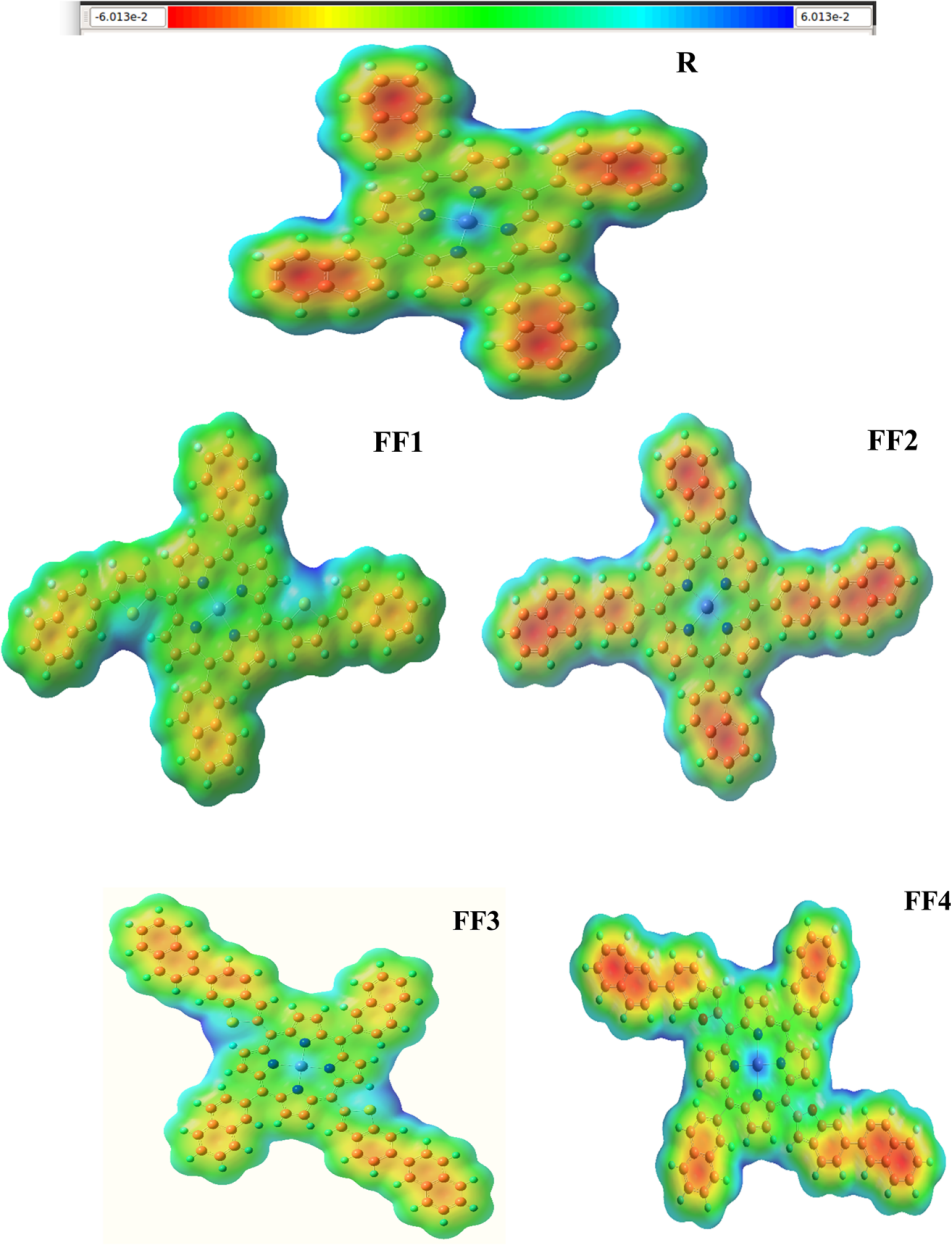
The electrostatic potential surfaces of the newly designed series (FF1–FF4) and the synthetic reference R.

The MEP analysis reveals dynamic charging sites and various charge separations and regions within the molecules. In molecules, each charge site or group of sites is often characterized by a distinct collection of data that delineates the molecule's unique properties. These components significantly influence the efficacy of molecular function.^49,50^Fig. 10 illustrates the outcomes of the computations for the MEP plot. The distinct characteristics of the molecules are discernible in the MEP plots after analyzing the color patterns and their combinations. A vibrant green segment of the molecule indicates neutrality, while blue signifies electropositivity. Conversely, the red hue highlights a detrimental area inside the cell. These color trends have been seen in both the R molecule and the designed compounds (FF1–FF4). An evaluation of the molecule's potential use in SC devices may be aided by the abundance of charge sites, which reveal its unique properties. All the possible uses of the materials are laid forth in these MEP diagrams. And because their MEP surfaces have a uniform charge distribution, the suggested molecules are better suited to the R. The results indicate that the PCE and consistency of organic solar cells may be enhanced by molecular side-chain modifications to develop superior molecules for future applications.^51^

### 3.9 Open circuit voltage

**Fig. 11:**
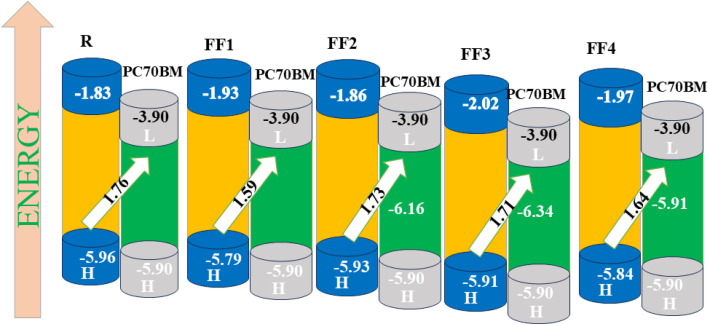
Calculated *V*_oc_ of the R molecule and design compounds (FF1–FF4) relative to PC70BM.

One of the most important parameters that determines how well organic solar cells (OSCs) work is their open circuit voltage (*V*_oc_). It exemplifies the maximum current that an optical device is capable of producing.^52^*V*_oc_ is influenced by several parameters, including energy levels, temperature, light intensity, light source, electrode function, charge transfer recombination, and ambient conditions. The *V*_oc_ is also contingent on the current saturation and light intensity, which facilitates device recombination. The disparity in the HOMO/LUMO energies between the donor and acceptor, together with modifications to the pi-bridge in molecules, dictates *V*_oc_. The *V*_oc_ rose when the acceptor's LUMO level is elevated by pi-bridge modification, and the donor's HOMO level is reduced. Our research on R (particularly FF1–FF4) reveals that the HOMO energy levels are initially antithetical to the LUMO energy levels of the established acceptor polymeric material PCB70BM, as shown in Fig. 11. The *V*_oc_ values are calculated by eqn (12).^53^

12 *V*_oc_ = (HOMO_(FF3)_ − LUMO_(PC70BM)_) − 0.3

PCB70BM was chosen for its recognized and theoretically and empirically substantiated donating characteristics. Considering that our developed FF-series mostly comprises donor molecules, we used the suggested acceptor materials, together with the polymer acceptor PCB70BM, to form a donor–acceptor complex via π-bridge modification. We used eqn (12) to determine the *V*_oc_ values of the compounds, excluding extraneous effects and focusing on the donor HOMO and acceptor (PCB70BM) LUMO energies. The reference molecule (R) shows a *V*_oc_ of 1.76 V, calculated from the energy difference between the donor HOMO and the PCB70BM LUMO. Fig. 11 compares R with the designed molecules (FF1–FF4). The higher HOMO levels of FF-compounds relative to R explain the reduced *V*_oc_ values of created molecules FF1–FF4.

### 3.10 Fill factor

The fill factor (FF) is the most important factor in a solar device's PCE and is also very important for improving OSC performance. It is a key characteristic that can be determined primarily through open-circuit analysis using a donor molecule and an acceptor polymer (PC70BM). Afterward, we determined the values of R and the new molecules (FF1–FF4). Eqn (13) (ref. 54) is used to calculate the fill-factor.13
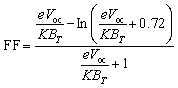


The aforementioned FF calculation demonstrates the normalized *V*_oc_ and a fundamental charge “*e*” equal to 1. Table 6 presents the computed *V*_oc_ and FF values. Both *V*_oc_ and FF have reached their optimal values for each of the modified molecules (FF1–FF4). The FF value is maximized for FF2 relative to other novel compounds, since it may be readily altered by including a π-spacer. When an FF is elevated, it generates excess power beyond its appropriate level. This indicates that charge carriers are recombining inside the device, hence reducing resistance losses. The elevated FF% of all suggested compounds (FF1–FF4) relative to R indicates that our design strategies were effective in creating photovoltaic materials suitable for OSCs.^55^

**Table 6:** Calculated FF%, open circuit voltage, and light-harvesting efficiency for FF1–FF4 molecules and reference (R)

Molecules	LHE in solvent	LHE in gas	*V*_oc_	FF	*E*_g_	% FF
R	0.980	0.897	1.76	0.9432	4.13	94.32
FF1	0.994	0.898	1.59	0.9399	3.86	93.98
FF2	0.994	0.898	1.73	0.9426	4.07	94.26
FF3	0.994	0.898	1.71	0.9423	3.89	94.22
FF4	0.999	0.900	1.64	0.9409	3.87	94.09

### 3.11 Light-harvesting efficiency analysis

Light harvesting can be used to produce charge carriers in optoelectronic materials. Additionally, it is believed that LHE and the *J*_sc_ of chemicals produced during device manufacture, as determined by eqn (14) & (15) (ref. 56) are closely related.14
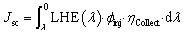
In this equation, *η*_collect_ denotes the efficacy of charge collecting, while *ϕ*_inj_ denotes the effectiveness of electron injection. The findings pertaining to the LHE phenomenon, which was investigated using eqn (15),^57^ are displayed in Table 6.

15 LHE = 1 – 10^−*f*^

The frequency of oscillation is denoted by “*f*” in eqn (15). Table 6 shows the LHE for R and the suggested compounds (FF1–FF4) in both gas and solvent (chlorobenzene) modes. FF4 had the highest LHE value (0.999) among the compounds designed in chlorobenzene solvent. This chemical may improve SC performance, as suggested. This exemplifies our efficient methodology for fabricating photovoltaic organic solar cell devices.

### 3.12 Natural population analysis

**Fig. 12:**
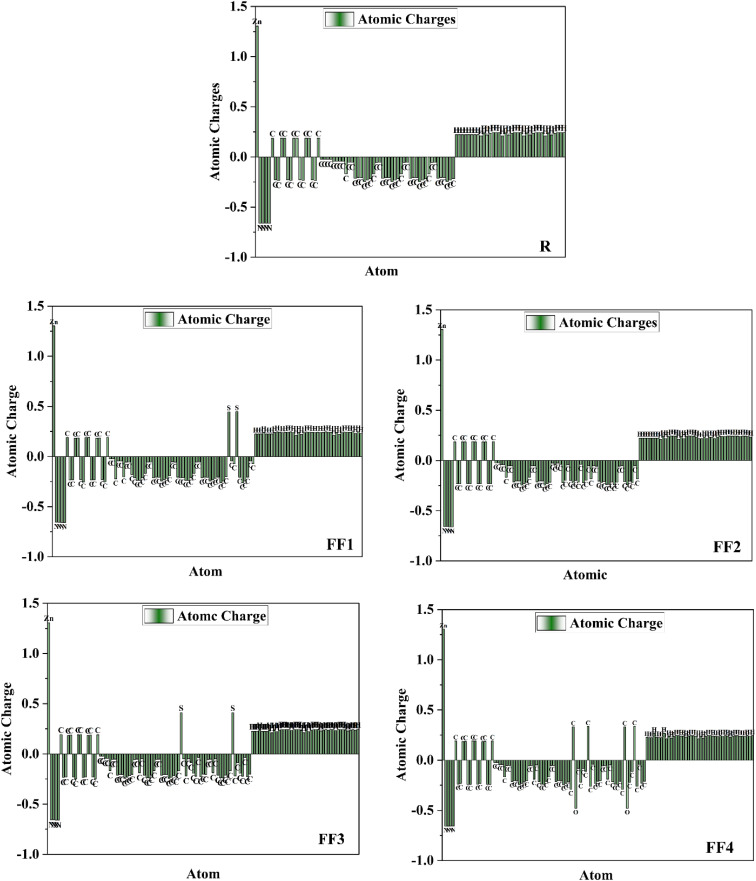
Plots of NPA atomic charges for all investigated porphyrin-based molecules along with reference R and design molecules (FF1–FF4).

Natural Population Analysis (NPA) is a method for determining atomic charges and electron distributions in molecular complexes. The NPA results in Fig. 12 show the net atomic charges for porphyrin-based molecules. Because they are close to carbon atoms, hydrogen atoms have a positive charge. Most of the carbon atoms in the donor and acceptor regions have negative charges, while the atoms that are bonded to sulfure have positive charges in FF1 and FF3. When nitrogen atoms are linked to a carbon atom, they usually have a negative charge. An oxygen atom acquires a negative charge when it combines with a positively charged carbon atom. In porphyrin-based complex high-throughput materials, negative charges are mostly found on carbon, nitrogen, and oxygen atoms, as well as all active centers (R and FF1–FF4). Making small changes to the acceptor units could further improve charge separation and enhance the device's overall performance.

### 3.13 Quadrupole analysis

**Fig. 13:**
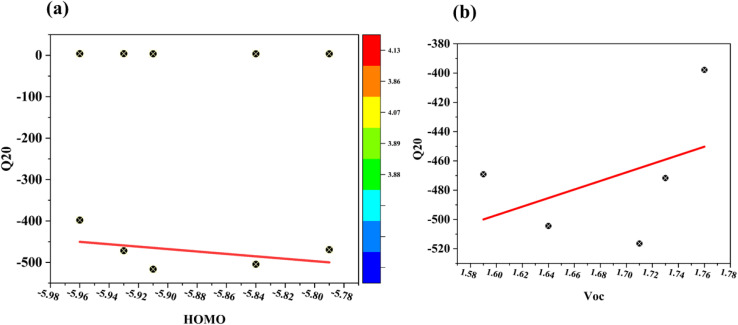
(a) Plot of the *Q*_20_–HOMO correlation for the investigated NFA complexes, where the color of each point represents the corresponding HOMO–LUMO gap. (b) Plot of the *Q*_20_–*V*_oc_ correlation for the investigated porphyrin-based complexes, with point color indicating the corresponding (LUMO + 1)–LUMO gap value.

*Q*_20_ is important for charge separation and recombination at the donor–acceptor interface, which directly affects short-circuit current and total power conversion efficiency in solar cells. The database analysis also highlighted the link between important computed parameters.^57^ Higher *Q*_20_ values result in increased *J*_sc_ output; however, excessive *Q*_20_ levels need to be optimized for both *J*_sc_ and *V*_oc_ since they lower the open-circuit voltage (*V*_oc_).^58^ The addition of end-capped acceptors to all developed (FF1–FF4) molecules results in improved quadrupole moment values and large HOMO/LUMO levels of energy at the same time, improving the device's performance (Fig. 13).

### 3.14 Charge transfer analysis between FF1 and PC70BM

**Fig. 14:**
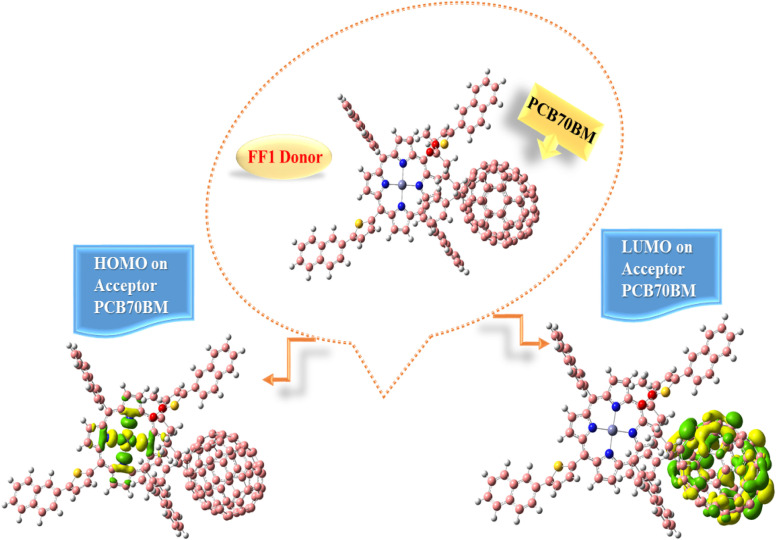
Electronic charge distribution of the FF1 donor–acceptor complex with PC70BM HOMO and LUMO.

The behavior of molecules has also been quantified in response to changes in their charges. The primary objective of this computation is to determine the behavior of our newly synthesized molecules as donors when interacting with the acceptor polymer. It was determined that FF1 performs effectively with the majority. The PC70BM acceptor polymer is widely utilized. The charge-transfer experiments with the polymer acceptor PC70BM utilized the donor FF1 due to its higher hole mobility. Mobility and charging gearbox efficiency. Compared with the other designed (FF1–FF4) molecules, FF1 had lower reorganizational holes and lower electron mobility. Parallel orientation of PC70BM and FF1 yields optimal alignment. The structure of this complex is illustrated in Fig. 14, following optimization using the CAM-B3LYP/6-31G(d,p) basis set. For optimal charge transfer at the interface, the donor–acceptor combination is constructed. Fig. 14 shows the distribution of the H–L pattern for PC70BM and FF1. The HOMO state was found in the middle of the donor segment of the molecule, and the LUMO state covered the entire acceptor region (PC70BM), resulting in an unanticipated distribution of the HOMO–LUMO states. The HOMO/LUMO patterns indicate a variation in charge density across molecules, which must have occurred at the donor–acceptor interface (FF1; PC70BM).

## 4 Conclusion

Finally, we carried out theoretical characterizations using advanced quantum-chemical techniques to investigate the photovoltaic, optoelectronic, and photophysical properties of the recently synthesized FF1–FF4 molecules for use in solar cells. We systematically synthesized four novel compounds, FF1–FF4, by modifying the π-spacer of the reference molecule R. All computations were conducted at the CAM-B3LYP/6-31G(d,p) theoretical level. The newly created molecules' calculated photophysical characteristics were systematically compared with those of the reference compound R. Additionally, FF1 exhibited a significant bathochromic shift upon dissolution, with a peak absorption wavelength (*λ*_max_) of 435 nm in the gas phase and 457 nm in chlorobenzene. The lowest excitation energy is found in the gas phase at 2.846 eV and in chloroform at 2.708 eV. Notably, out of all the molecules that have been made, FF1 has the smallest energy gap, measuring only 3.86 eV. These specially designed molecules (FF1–FF4) with effective pi-bridge modification improved charge distribution, resulting in a higher number of LUMOs on the acceptor units. Also, new effective acceptor molecular orbitals close the gaps between HOMO and LUMO while increasing the total charge density. The smaller value of *λ*_*e*_ in all four of the new molecules (FF1–FF4) shows that the electrons can move their charges around more easily. These new compounds are interesting options for OSCs because they have better optoelectronic qualities than the standard reference (R) compound.

## Conflicts of interest

All authors have stated that they have no conflicts of interest.

## Data Availability

Data supporting the findings above will be available upon request. All enquiries should be addressed to the author.
